# Supplementary material for: Defective ventral neurogenesis due to midfetal Chd8 mutation drives autistic-like behavior in mice
Source: Nat Commun. 2026 May 27;17:4457. doi: 10.1038/s41467-026-73416-2 (PMC13216556; doi:10.1038/s41467-026-73416-2)
Supplement: Supplementary file 1 — Supplementary Information [file 41467_2026_73416_MOESM1_ESM.pdf]

Supplementary Information

Defective ventral neurogenesis due to midfetal *Chd8* mutation drives autistic-like behavior in mice

Kenta Nitahara, Atsuki Kawamura, Ayumu Tashiro, Tomoya Iwasaki, Shin-Ichi Horike, Jumpei Terakawa, Takiko Daikoku, Koichi Higashi, Ken Kurokawa, Kiyoko Kato & Masaaki Nishiyama

Supplementary Figures 1–15

Supplementary Data 1–3

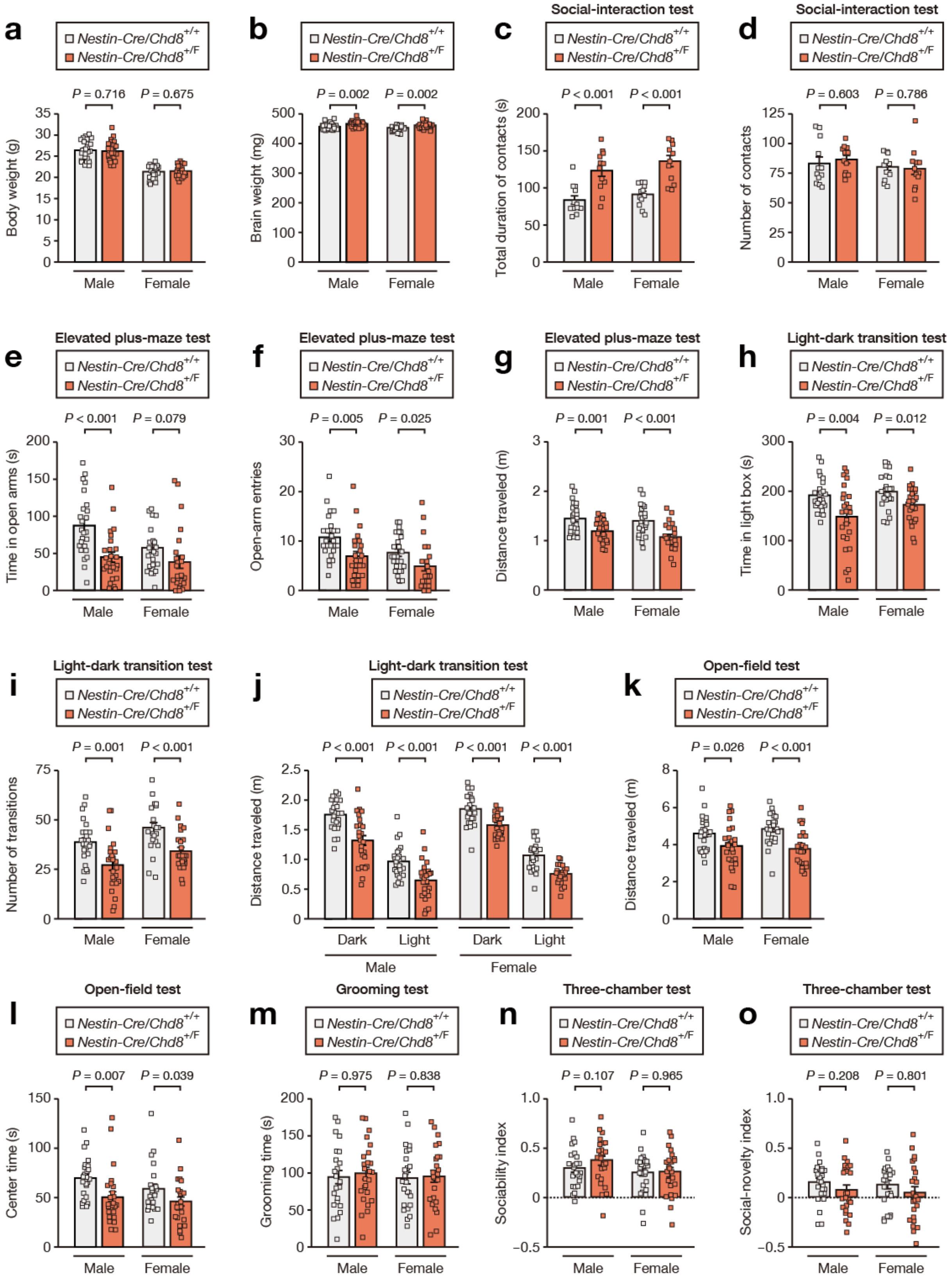

a
Nestin-Cre/Chd8+/+
Nestin-Cre/Chd8+/F
P = 0.716
P = 0.675
Body weight (g)
Male
Female
b
P = 0.002
P = 0.002
Brain weight (mg)
c
Social-interaction test
P < 0.001
P < 0.001
Total duration of contacts (s)
d
Social-interaction test
P = 0.603
P = 0.786
Number of contacts
e
Elevated plus-maze test
P < 0.001
P = 0.079
Time in open arms (s)
f
Elevated plus-maze test
P = 0.005
P = 0.025
Open-arm entries
g
Elevated plus-maze test
P = 0.001
P < 0.001
Distance traveled (m)
h
Light-dark transition test
P = 0.004
P = 0.012
Time in light box (s)
i
Light-dark transition test
P = 0.001
P < 0.001
Number of transitions
j
Light-dark transition test
P < 0.001
P < 0.001
P < 0.001
P < 0.001
Distance traveled (m)
Dark
Light
Dark
Light
Male
Female
k
Open-field test
P = 0.026
P < 0.001
Distance traveled (m)
l
Open-field test
P = 0.007
P = 0.039
Center time (s)
m
Grooming test
P = 0.975
P = 0.838
Grooming time (s)
n
Three-chamber test
P = 0.107
P = 0.965
Sociability index
o
Three-chamber test
P = 0.208
P = 0.801
Social-novelty index

Supplementary Fig. 1 Neural stem cell–specific mutation of *Chd8* results in macrocephaly and behavioral abnormalities in female and male mice. **a**,**b**, Body weight (**a**) and brain weight (**b**) for mice subjected to behavioral tests. **c**,**d**, Total duration of contacts (**c**) and total number of contacts (**d**) for the social-interaction test. **e**–**g**, Time spent in the open arms (**e**), number of entries into the open arms (**f**), and total distance traveled (**g**) for the elevated plus-maze test. **h**–**j**, Time spent in the light chamber (**h**), number of transitions between the light and dark chambers (**i**), and total distance traveled in the light or dark chamber (**j**) for the light-dark transition test. **k**,**l**, Total distance traveled (**k**) and time spent in the central area (**l**) for the open-field test. **m**, Total duration of self-grooming in the self-grooming test. **n**,**o**, Scores for the sociability index (**n**) and social-novelty index (**o**) in three-chamber tests. All data are means ± s.e.m. and were obtained with *Nestin-Cre*/*Chd8*$^{+/+}$ and *Nestin-Cre*/*Chd8*$^{+/\mathrm{F}}$ male and female mice. For the social-interaction test only, n = 12 pairs per genotype (one pair = two unfamiliar mice), with n = 25 mice per genotype for all other tests. All behavioral tests were conducted with mice at 9 to 13 weeks of age. P values were calculated with the two-tailed Student's t test.

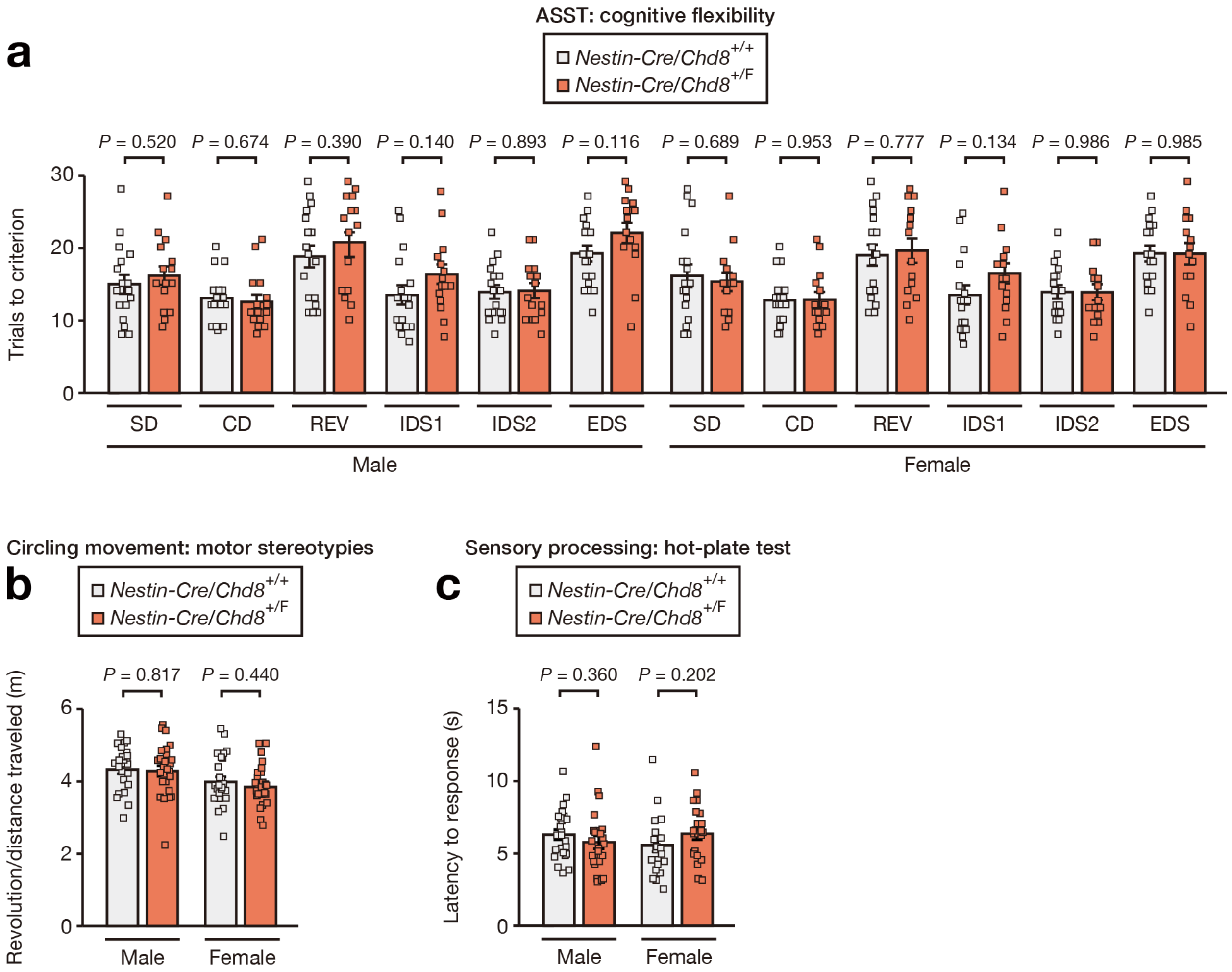

Supplementary Fig. 2 Neural stem cell–specific mutation of *Chd8* does not affect cognitive flexibility, motor stereotypies, or sensory processing in female and male mice. a, Number of trials required to reach the criterion (six consecutive correct choices) for SD, CD, REV, IDS1, IDS2, and EDS in the attentional set-shifting task (ASST). SD, simple discrimination; CD, compound discrimination; REV, reversal; IDS, intradimensional shift; EDS, extradimensional shift. **b**, Number of perimeter circling behaviors divided by the total distance traveled for the open-field test. **c**, Latency to the first nocifensive response in the hot-plate test. All data are means ± s.e.m. and were obtained with *Nestin-Cre*/*Chd8*$^{+/+}$ and *Nestin-Cre*/*Chd8*$^{+/F}$ male (n = 17 and 15 per each genotype for ASST, n = 25 per genotype for circling movement, and n = 24 per genotype for hot-plate test) and female (n = 17 and 14 per each genotype for ASST, n = 25 per

genotype for circling movement, and *n* = 22 per genotype for hot-plate test) mice. All behavioral tests were conducted with mice at 9 to 13 weeks of age. *P* values were calculated with the two-tailed Student's *t* test.

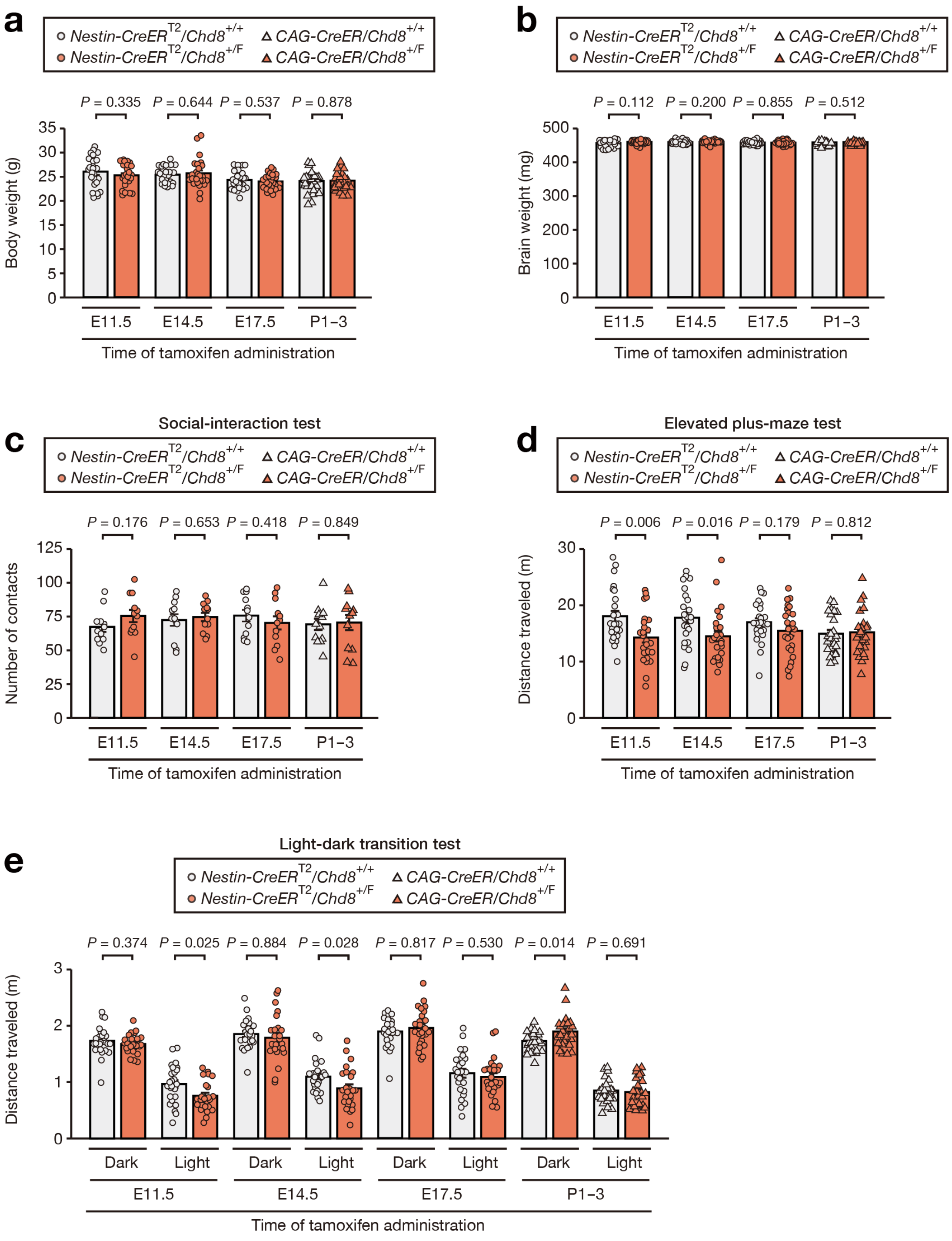

Supplementary Fig. 3 Identification of the critical period for the development of autistic-like behavior due to *Chd8* heterozygous mutation in male mice. a,**b**, Body weight (**a**) and

brain weight (**b**) for mice subjected to behavioral tests. **c**, Total number of contacts for the social-interaction test. **d**, Total distance traveled in the elevated plus-maze test. **e**, Total distance traveled in the light or dark chamber for the light-dark transition test. All data are means ± s.e.m. and were obtained from *Nestin-CreER*^{T2}/*Chd8*$^{+/+}$ and *Nestin-CreER*^{T2}/*Chd8*$^{+/F}$ adult male mice treated with tamoxifen at E11.5, E14.5, or E17.5, or from *CAG-CreER*/*Chd8*$^{+/+}$ and *CAG-CreER*/*Chd8*$^{+/F}$ adult male mice treated with tamoxifen at P1–3. For the social-interaction test only, n = 12 pairs per genotype per induction time (one pair = two unfamiliar mice), with n = 25 mice per genotype per induction time for all other tests. All behavioral examinations were conducted with male mice at 9 to 13 weeks of age. *P* values are calculated with the two-tailed Student's *t* test.

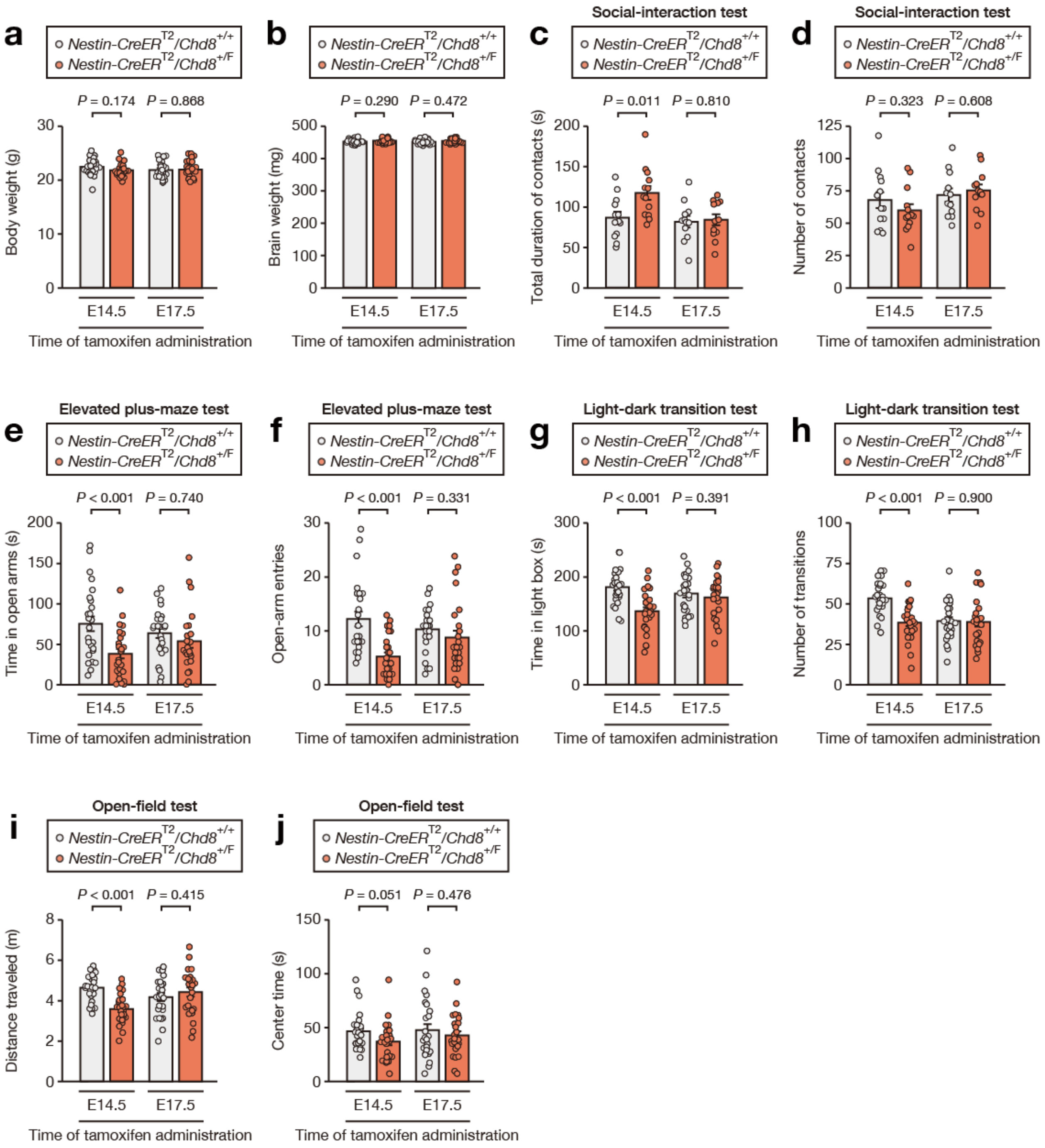

Supplementary Fig. 4 Identification of the critical period for the development of autistic-like behavior due to *Chd8* heterozygous mutation in female mice. **a**,**b**, Body weight (**a**) and brain weight (**b**) for mice subjected to behavioral tests. **c**,**d**, Total duration of contacts (**c**) and

total number of contacts (**d**) for the social-interaction test. **e**,**f**, Time spent in the open arms (**e**) and number of entries into the open arms (**f**) for the elevated plus-maze test. **g**,**h**, Time spent in the light chamber (**g**) and number of transitions between the light and dark chambers (**h**) for the light-dark transition test. **i**,**j**, Total distance traveled (**i**) and time spent in the central area (**j**) for the open-field test. All data are means ± s.e.m. and were obtained with *Nestin-CreER*^{T2}/*Chd8*$^{+/+}$ and *Nestin-CreER*^{T2}/*Chd8*$^{+/F}$ adult female mice treated with tamoxifen at E14.5 (n = 26 mice per genotype with the exception that n = 13 pairs of mice for the social-interaction test) or E17.5 (n = 25 mice per genotype, with the exception that n = 12 pairs of mice for the social-interaction test). All behavioral tests were conducted with female mice at 9 to 13 weeks of age. P values were calculated with the two-tailed Student's t test.

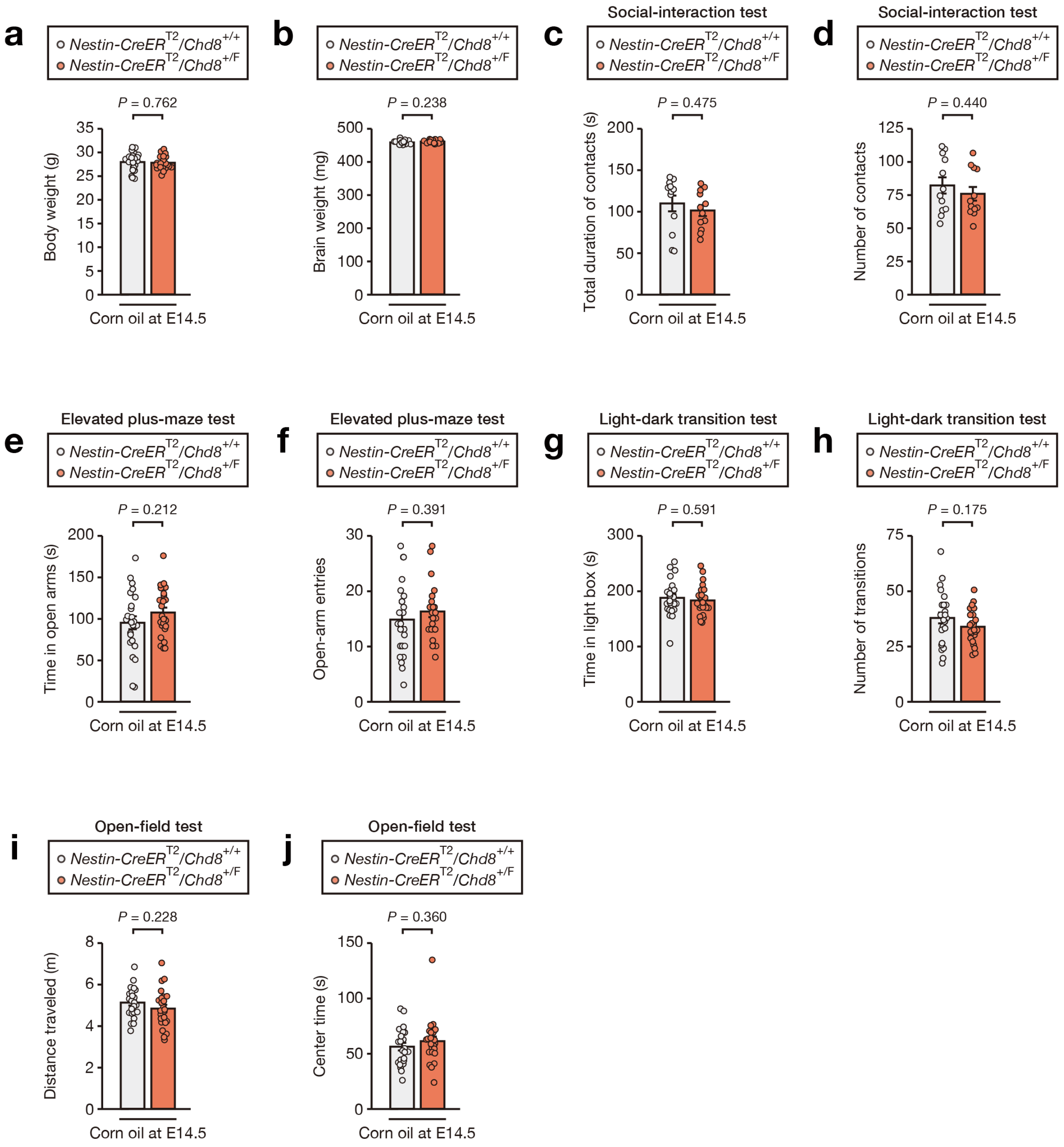

Supplementary Fig. 5 Procedures of cesarean section and fostering were not associated with behavioral phenotypes. a,**b**, Body weight (**a**) and brain weight (**b**) for mice subjected to behavioral tests. **c**,**d**, Total duration of contacts (**c**) and total number of contacts (**d**) for the social-interaction test. **e**,**f**, Time spent in the open arms (**e**) and number of entries into the open arms (**f**)

for the elevated plus-maze test. **g**,**h**, Time spent in the light chamber (**g**) and number of transitions between the light and dark chambers (**h**) for the light-dark transition test. **i**,**j**, Total distance traveled (**i**) and time spent in the central area (**j**) for the open-field test. All data are means ± s.e.m. and were obtained with *Nestin-CreER*$^{\mathrm{T2}}$/*Chd8*$^{+/+}$ and *Nestin-CreER*$^{\mathrm{T2}}$/*Chd8*$^{+/\mathrm{F}}$ male mice (n = 24 mice per genotype, with the exception that n = 12 pairs of mice for the social-interaction test) that underwent administration of corn oil at E14.5 and subsequent delivery by cesarean section and fostering at E19.5. All behavioral tests were conducted with male mice at 9 to 13 weeks of age. P values were calculated with the two-tailed Student's t test.

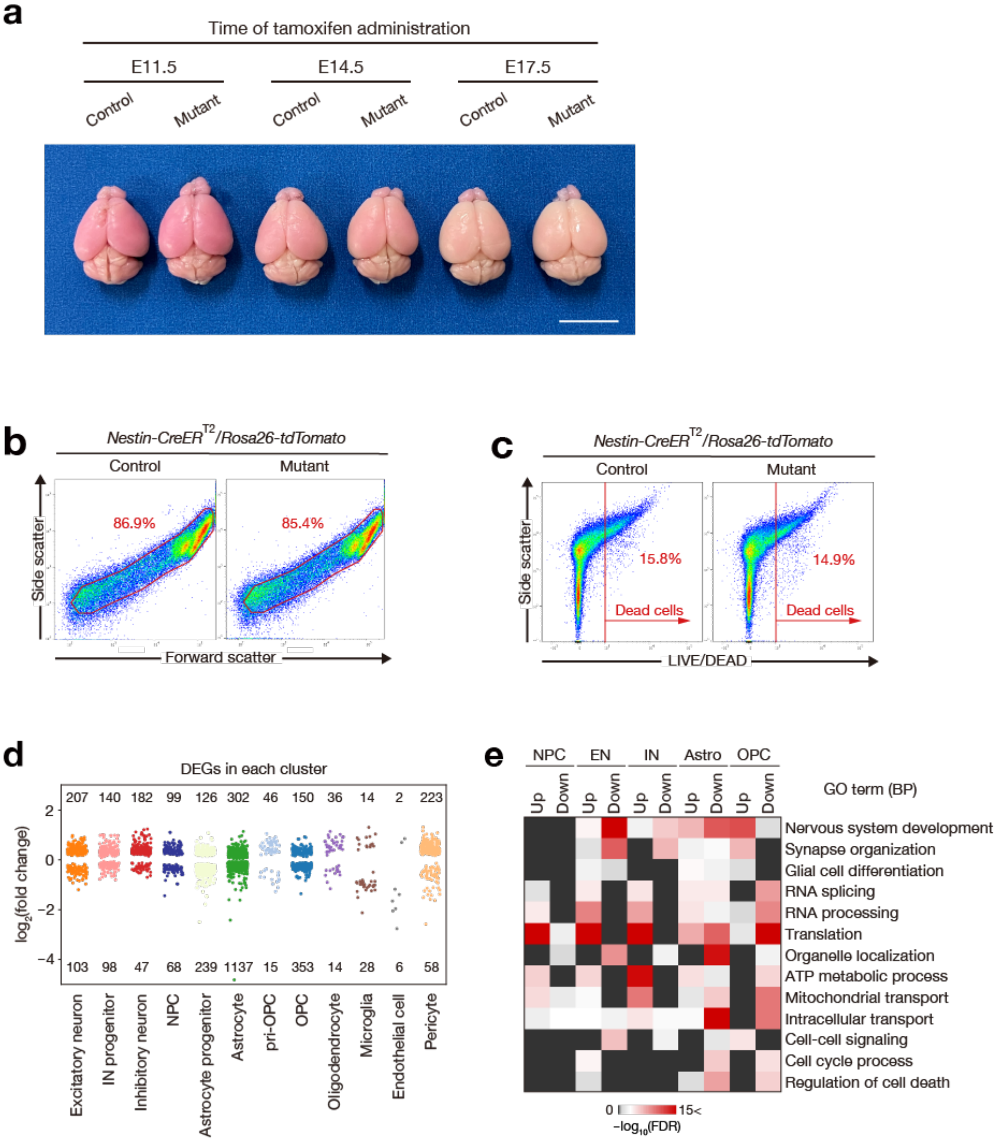

Supplementary Fig. 6 Combination of lineage tracing and scRNA-seq analysis reveals cell type–specific alterations in gene expression associated with midfetal induction of *Chd8* heterozygous mutation. a, Appearance of the brain of *Nestin-CreER*^{T2}*/Rosa26-tdTomato/Chd8*$^{+/+}$ (control) or *Nestin-CreER*^{T2}*/Rosa26-tdTomato/Chd8*$^{+/F}$ (mutant) mice at 8 weeks of age after tamoxifen administration at E11.5, E14.5, or E17.5. Scale bar, 1 cm. **b**,**c**, FACS strategy of gating for forward and side scatter (**b**) and for Pacific Blue fluorescence (**c**) to exclude doublets/debris and dead cells, respectively. **d**, Fold change in expression for DEGs in

each cluster of mutant cells compared with control cells based on scRNA-seq data. The numbers of up- and downregulated DEGs are shown in the upper and lower parts of the plot, respectively. **e**, Gene Ontology (GO) analysis for DEGs whose expression was up- or downregulated in the indicated clusters. EN, excitatory neuron; IN, inhibitory neuron; Astro, astrocyte; BP, biological process.

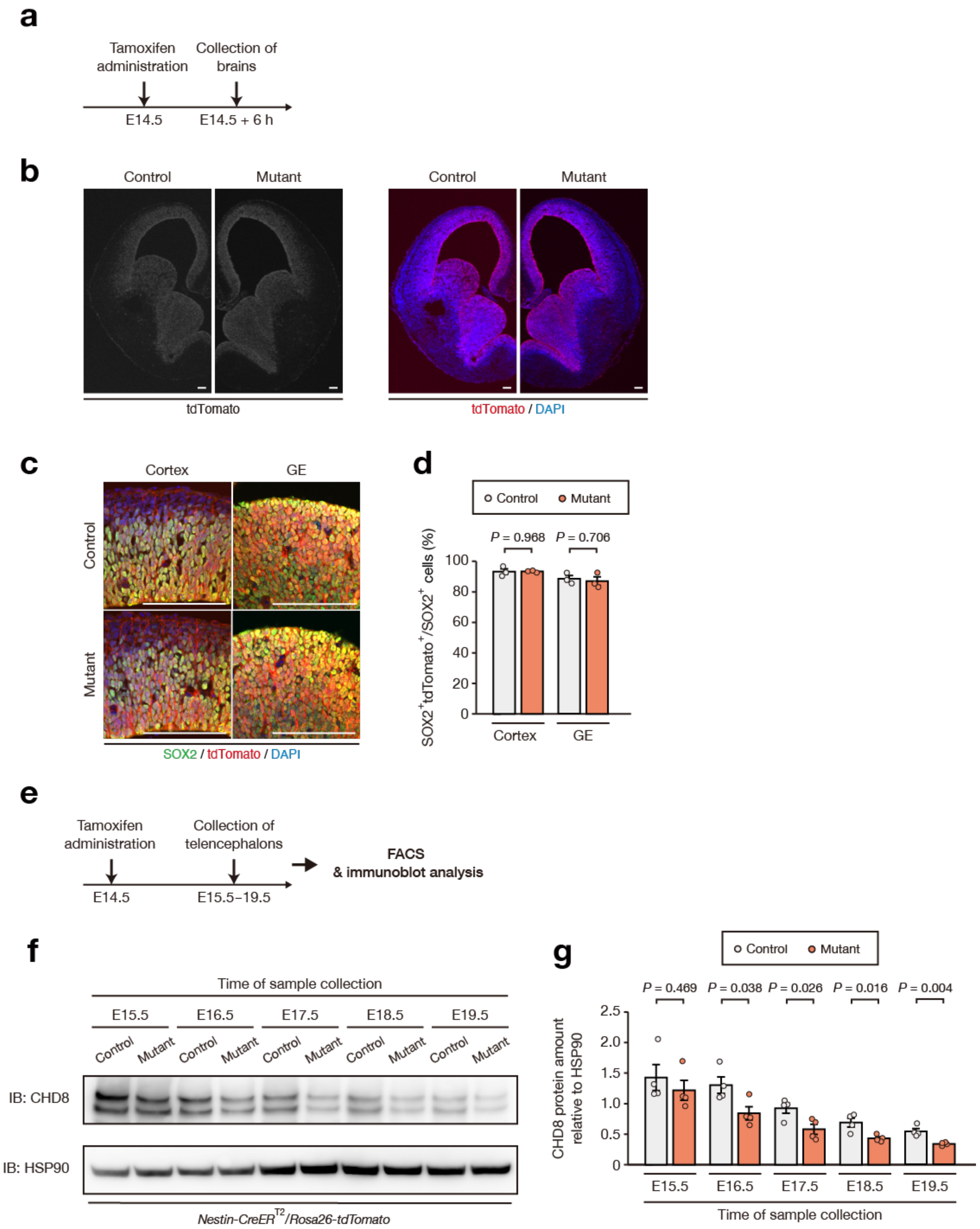

a
Tamoxifen administration
Collection of brains
E14.5
E14.5 + 6 h
b
Control
Mutant
tdTomato
tdTomato / DAPI
c
Cortex
GE
Control
Mutant
SOX2 / tdTomato / DAPI
d
Control
Mutant
P = 0.968
P = 0.706
SOX2+tdTomato+/SOX2+ cells (%)
Cortex
GE
e
Tamoxifen administration
Collection of telencephalons
E14.5
E15.5–19.5
FACS & immunoblot analysis
f
Time of sample collection
E15.5
E16.5
E17.5
E18.5
E19.5
Control
Mutant
IB: CHD8
IB: HSP90
Nestin-CreERT2/Rosa26-tdTomato
g
Control
Mutant
P = 0.469
P = 0.038
P = 0.026
P = 0.016
P = 0.004
CHD8 protein amount relative to HSP90
E15.5
E16.5
E17.5
E18.5
E19.5
Time of sample collection

Supplementary Fig. 7 *Chd8* heterozygous mutation is associated with accelerated cell-cycle exit and differentiation in ventral progenitor cells. a–**d**, Protocol for tamoxifen administration and sample collection (**a**), immunofluorescence staining of tdTomato (**b**) or of SOX2 and tdTomato (**c**), and quantification of the number of cells positive for tdTomato among $SOX2^+$ cells in the cortex and ganglionic eminence (GE) (**d**) for *Nestin-CreER*^{T2}*/Rosa26-tdTomato/Chd8*$^{+/+}$ (control) and *Nestin-CreER*^{T2}*/Rosa26-tdTomato/Chd8*$^{+/F}$ (mutant) embryos (*n* = 3 mice per genotype). Scale bars, 100 µm. **e**–**g**, Protocol for tamoxifen administration and sample collection (**e**) as well as immunoblot (IB) analysis of CHD8 and HSP90 (loading control) (**f**) and quantification of the CHD8/HSP90 band intensity ratio (**g**) for tdTomato-positive cells isolated by FACS from *Nestin-CreER*^{T2}*/Rosa26-tdTomato/Chd8*$^{+/+}$ (control) and *Nestin-CreER*^{T2}*/Rosa26-tdTomato/Chd8*$^{+/F}$ (mutant) embryos (*n* = 4 mice per genotype). All quantitative data are means ± s.e.m. *P* values were calculated with the two-tailed Student's *t* test.

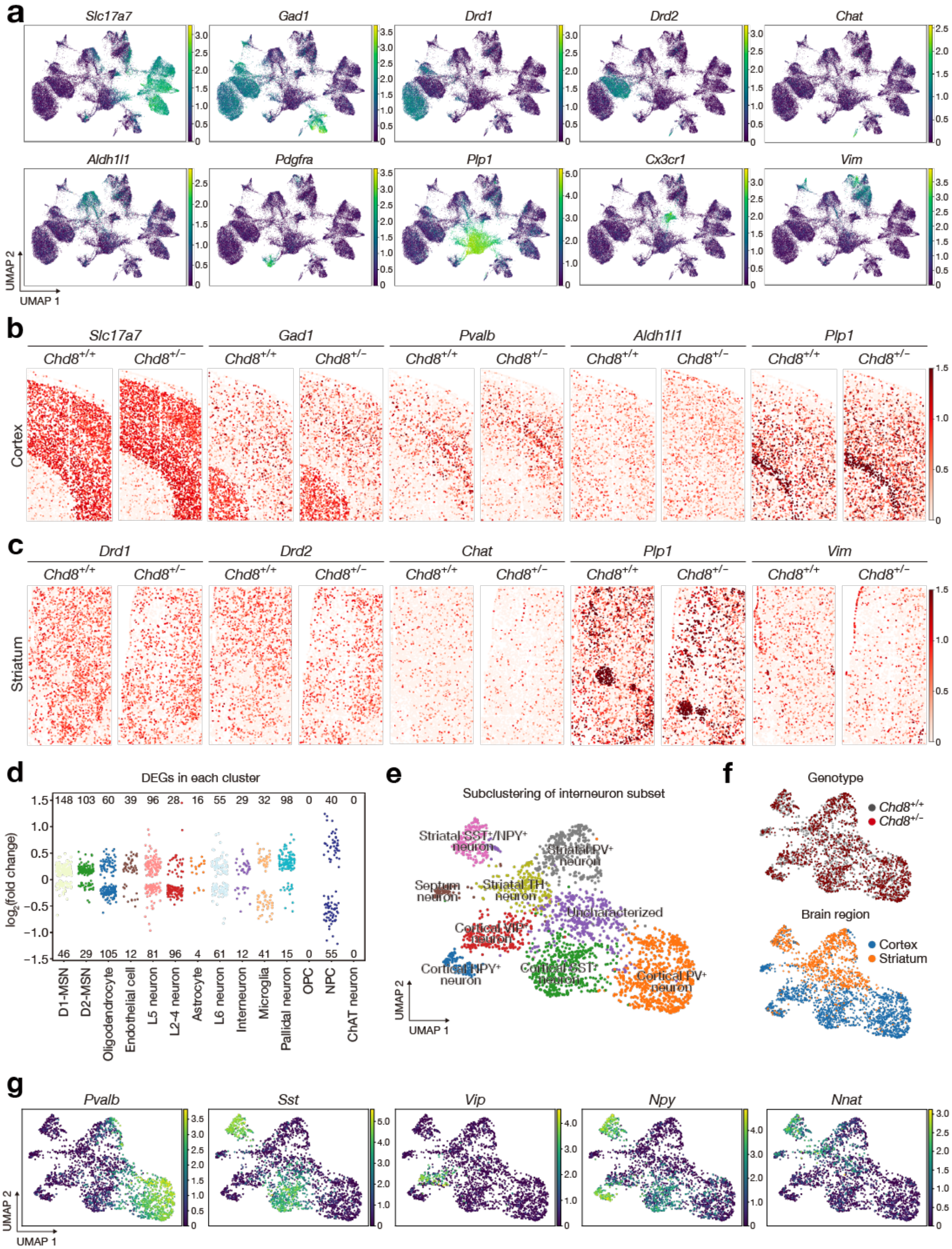

a
Slc17a7
Gad1
Drd1
Drd2
Chat
Aldh1l1
Pdgfra
Plp1
Cx3cr1
Vim
UMAP 1
UMAP 2
b
Slc17a7
Gad1
Pvalb
Aldh1l1
Plp1
Chd8+/+
Chd8+/−
Cortex
c
Drd1
Drd2
Chat
Plp1
Vim
Striatum
d
DEGs in each cluster
log2(fold change)
148 103 60 39 96 28 16 55 29 32 98 0 40 0
46 29 105 12 81 96 4 61 12 41 15 0 55 0
D1-MSN
D2-MSN
Oligodendrocyte
Endothelial cell
L5 neuron
L2-4 neuron
Astrocyte
L6 neuron
Interneuron
Microglia
Pallidal neuron
OPC
NPC
ChAT neuron
e
Subclustering of interneuron subset
Striatal SST+/NPY+ neuron
Striatal PV+ neuron
Septum neuron
Striatal TH neuron
Uncharacterized
Cortical VIP+ neuron
Cortical NPY+ neuron
Cortical SST+ neuron
Cortical PV+ neuron
f
Genotype
Chd8+/+
Chd8+/−
Brain region
Cortex
Striatum
g
Pvalb
Sst
Vip
Npy
Nnat

Supplementary Fig. 8 Spatial transcriptome analysis reveals that *Chd8* mutation is associated with region-specific changes in gene expression in the adult brain. a, Heat maps for expression levels of the indicated cell marker genes shown on the UMAP for spatial transcriptome analysis. **b**,**c**, Spatial distribution for expression levels of the indicated cell marker genes in the cortex (**b**) and striatum (**c**) of mutant mice compared with control mice. **d**, Fold change in expression for DEGs in each cluster of mutant mice compared with control mice based on spatial transcriptome data. The numbers of up- or downregulated DEGs are indicated in the upper and lower parts of the plot, respectively. **e**, UMAP showing nine subclusters for the interneuron cluster in Figure 4b. **f**, Distribution of genotype (upper panel) and brain region (lower panel) shown on the UMAP of the interneuron cluster. **g**, Heat maps for expression levels of the indicated cell marker genes shown on the UMAP of the interneuron cluster.

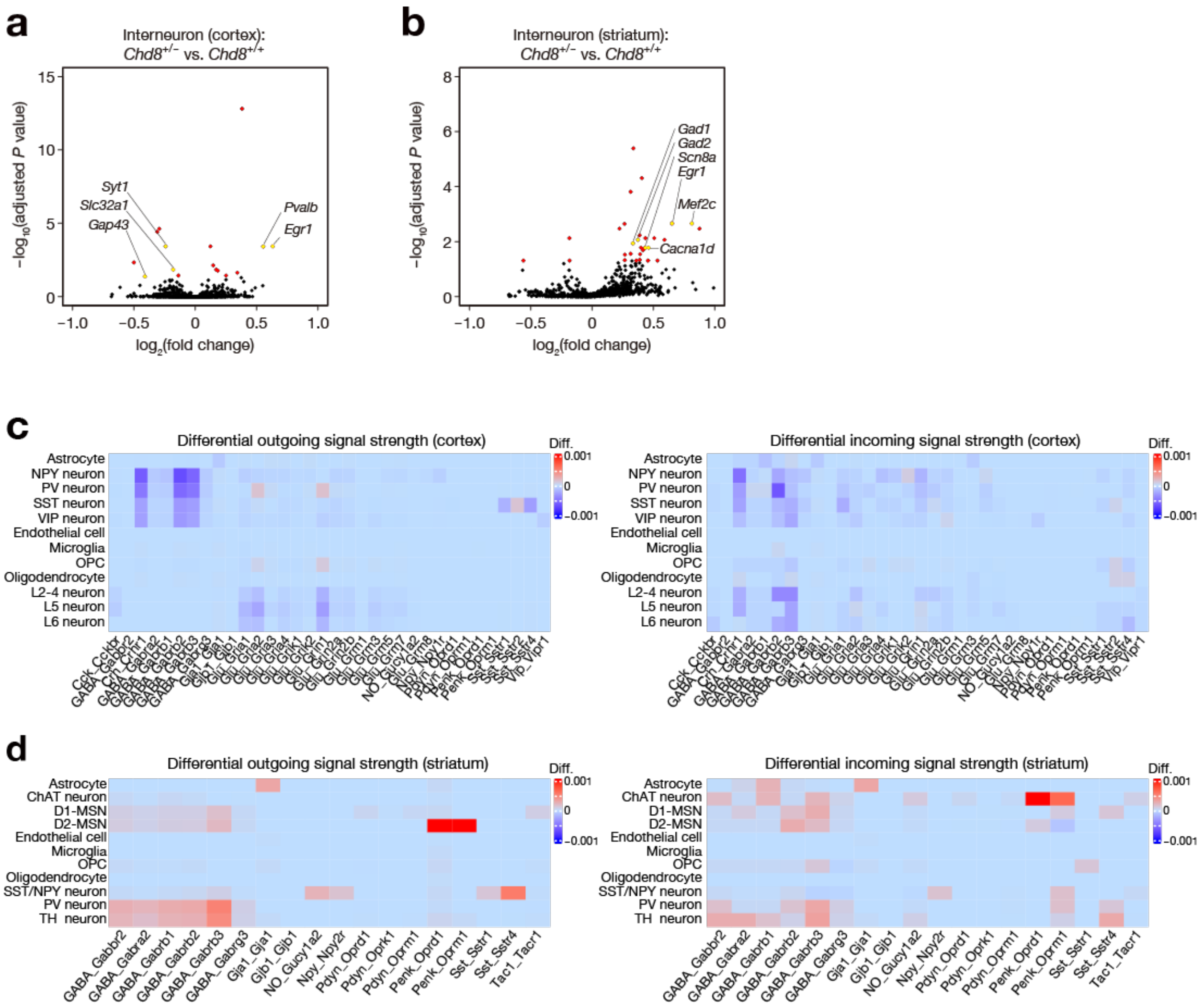

Supplementary Fig. 9 Spatial transcriptome analysis reveals that *Chd8* mutation is associated with region-specific changes in gene expression and cell communication in the adult brain. a,**b**, Volcano plots for DEGs in the interneuron cluster for the cortex (**a**) and striatum (**b**) of mutant mice compared with control mice. DEGs (FDR-adjusted *P* value of <0.05) are shown in red. Synapse-, neurotransmitter receptor–, and neuronal activity–related genes are highlighted in yellow. **c**,**d**, Heat maps based on NeuronChat analysis for the differential outgoing (left panels) and incoming (right panels) signal strengths across cell types are shown for the cortex (**c**) and striatum (**d**) of mutant mice compared with control mice.

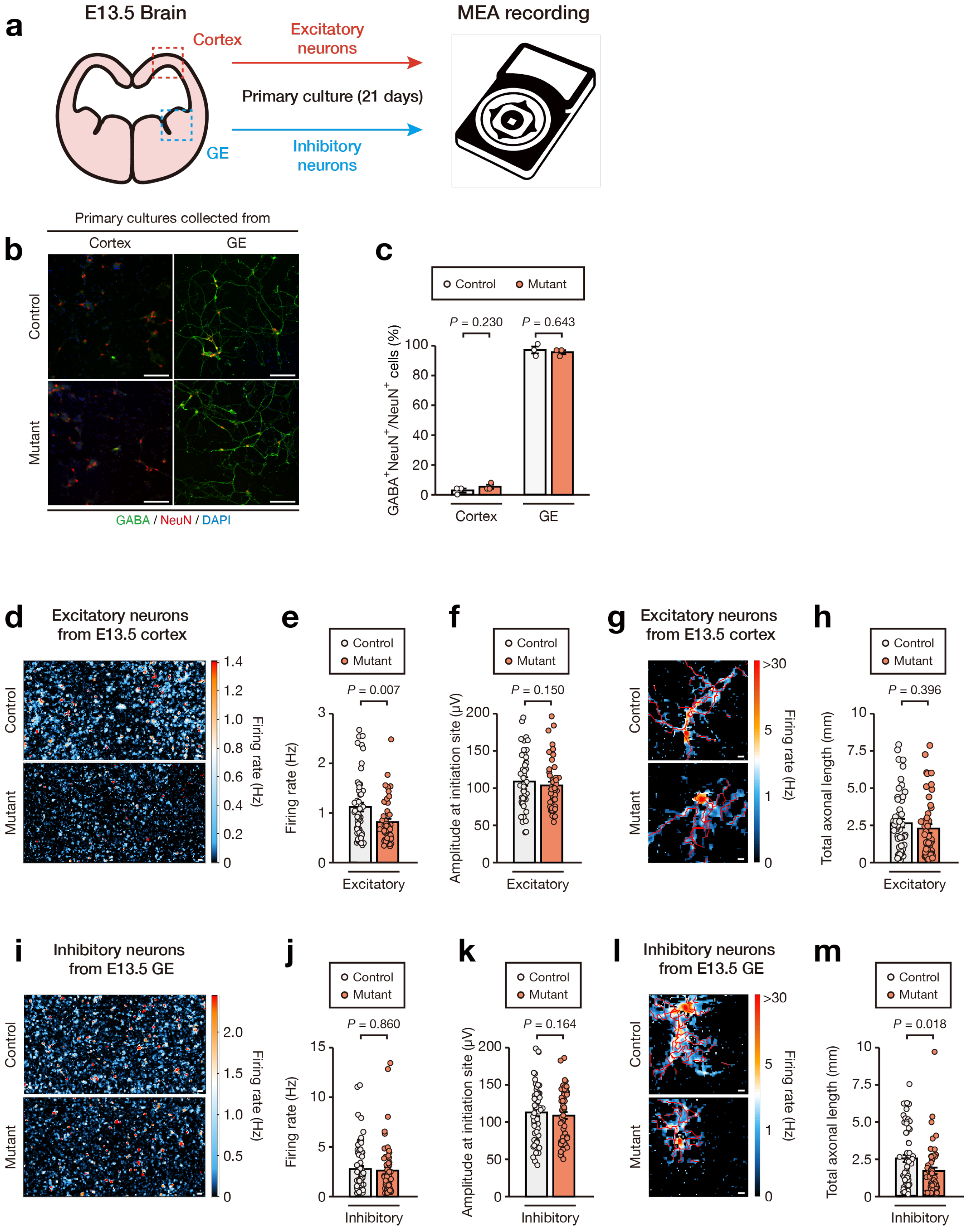
a
E13.5 Brain
Cortex
GE
Excitatory neurons
Primary culture (21 days)
Inhibitory neurons
MEA recording
b
Primary cultures collected from
Cortex
GE
Control
Mutant
GABA / NeuN / DAPI
c
Control
Mutant
P = 0.230
P = 0.643
GABA+NeuN+/NeuN+ cells (%)
Cortex
GE
d
Excitatory neurons from E13.5 cortex
Firing rate (Hz)
e
P = 0.007
Firing rate (Hz)
Excitatory
f
P = 0.150
Amplitude at initiation site (μV)
Excitatory
g
Excitatory neurons from E13.5 cortex
>30
Firing rate (Hz)
h
P = 0.396
Total axonal length (mm)
Excitatory
i
Inhibitory neurons from E13.5 GE
Firing rate (Hz)
j
P = 0.860
Firing rate (Hz)
Inhibitory
k
P = 0.164
Amplitude at initiation site (μV)
Inhibitory
l
Inhibitory neurons from E13.5 GE
>30
Firing rate (Hz)
m
P = 0.018
Total axonal length (mm)
Inhibitory

Supplementary Fig. 10 Microelectrode array recordings reveal impaired function of *Chd8*$^{+/–}$ mouse neurons. a, Strategy for evaluation of excitatory and inhibitory neurons by MEA analysis. Primary cultures of excitatory or inhibitory neurons were prepared from the E13.5 mouse cortex and ganglionic eminence (GE), respectively, and were subjected to MEA recording. **b**,**c**, Immunofluorescence staining of GABA and NeuN (**b**) as well as quantification of the number of cells positive for GABA among NeuN^{+} cells (**c**) in the cultures of primary neurons established from the E13.5 cortex or GE of *Chd8*$^{+/+}$ (control) and *Chd8*$^{+/–}$ (mutant) mice (*n* = 3 cultures per genotype). Scale bars, 100 μm. **d**–**f**, Spatial heat maps of firing rate for each electrode unit across the MEAs (**d**) as well as quantification of neuronal firing rate (**e**) and spike amplitude at the initiation site (**f**) for cultures prepared from the E13.5 cortex (excitatory neurons) of mutant and control mice (*n* = 52 neurons for control, *n* = 48 neurons for mutant). Scale bars, 100 μm. **g**,**h**, Heat maps of firing rate on the MEAs, with the reconstructed axonal trajectory shown in red (**g**), as well as quantification of total axonal length for neurons (**h**) in cultures prepared from the E13.5 cortex of mutant and control mice (*n* = 52 neurons for control, *n* = 48 neurons for mutant). Scale bars, 100 μm. **i**–**k**, Spatial heat maps of firing rate for each electrode unit across the MEAs (**i**) as well as quantification of neuronal firing rate (**j**) and spike amplitude at the initiation site (**k**) for cultures prepared from the E13.5 GE (inhibitory neurons) of mutant and control mice (*n* = 63 neurons for control, *n* = 56 neurons for mutant). Scale bars, 100 μm. **l**,**m**, Heat maps of firing rate on the MEAs, with the reconstructed axonal trajectory shown in red (**l**), as well as quantification of total axonal length for neurons (**m**) in cultures prepared from the E13.5 GE of mutant and control mice (*n* = 63 neurons for control, *n* = 56 neurons for mutant). Scale bars, 100 μm. All quantitative data are means ± s.e.m., and the *P* values were calculated with the two-tailed Student's *t* test.

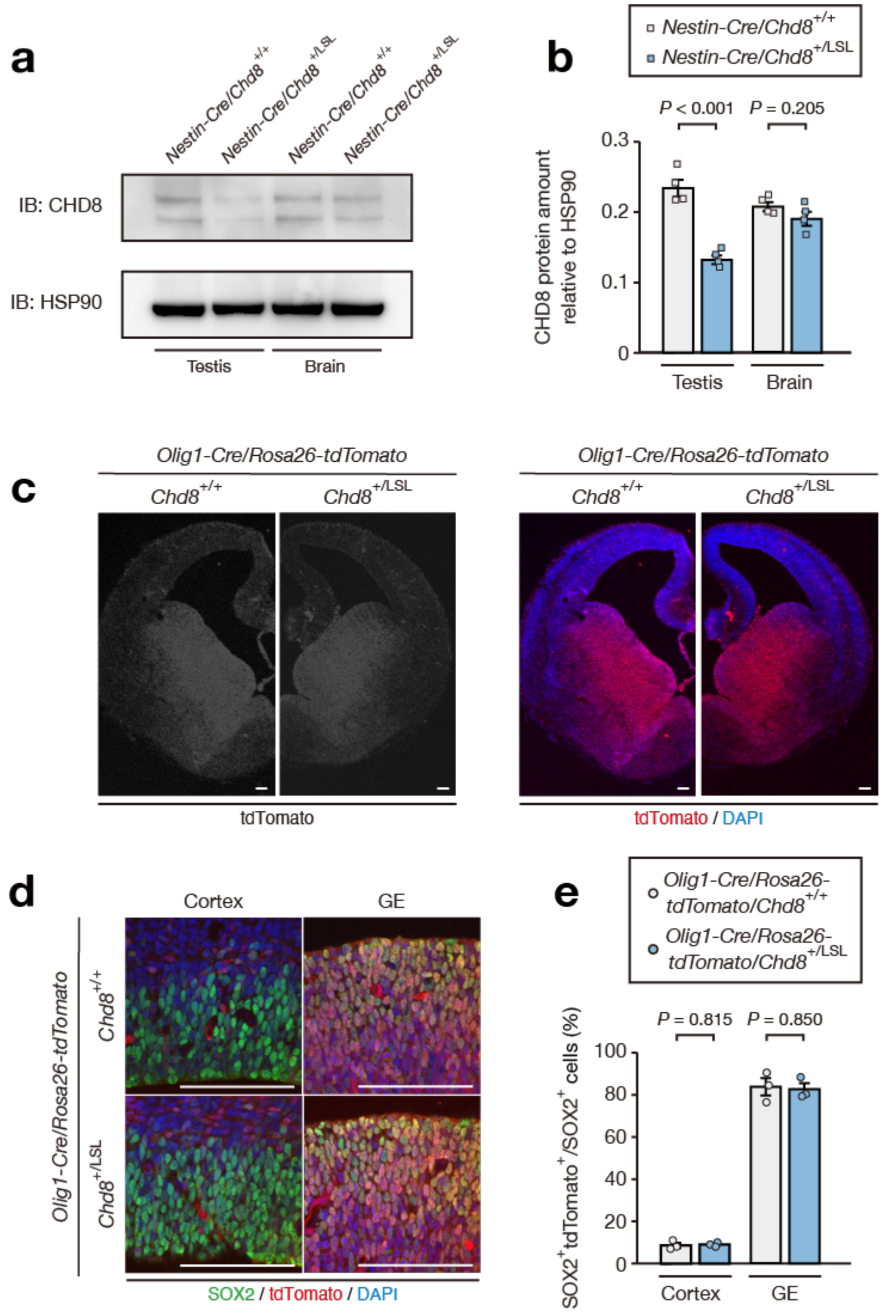

Supplementary Fig. 11 Genetic rescue of *Chd8* expression in neural stem cells and localization of *Olig1-Cre* expression for ventral progenitor cells. **a**,**b**, Immunoblot analysis of CHD8 and HSP90 (loading control) (**a**) as well as quantification of the CHD8/HSP90 band intensity ratio (**b**) for testis and brain of mice of the indicated genotypes at 8 weeks of age ($n = 4$

mice per genotype). **c**–**e**, Immunofluorescence staining of tdTomato (**c**) and of SOX2 and tdTomato (**d**), as well as quantification of the number of cells positive for tdTomato among $SOX2^+$ cells (**e**), for the cortex and ganglionic eminence (GE) of *Olig1-Cre/Rosa26-tdTomato/Chd8*$^{+/+}$ and *Olig1-Cre/Rosa26-tdTomato/Chd8*$^{+/LSL}$ embryos at E14.5 (*n* = 3 mice per genotype). Scale bars, 100 µm. All quantitative data are means ± s.e.m. *P* values were calculated with the two-tailed Student's *t* test.

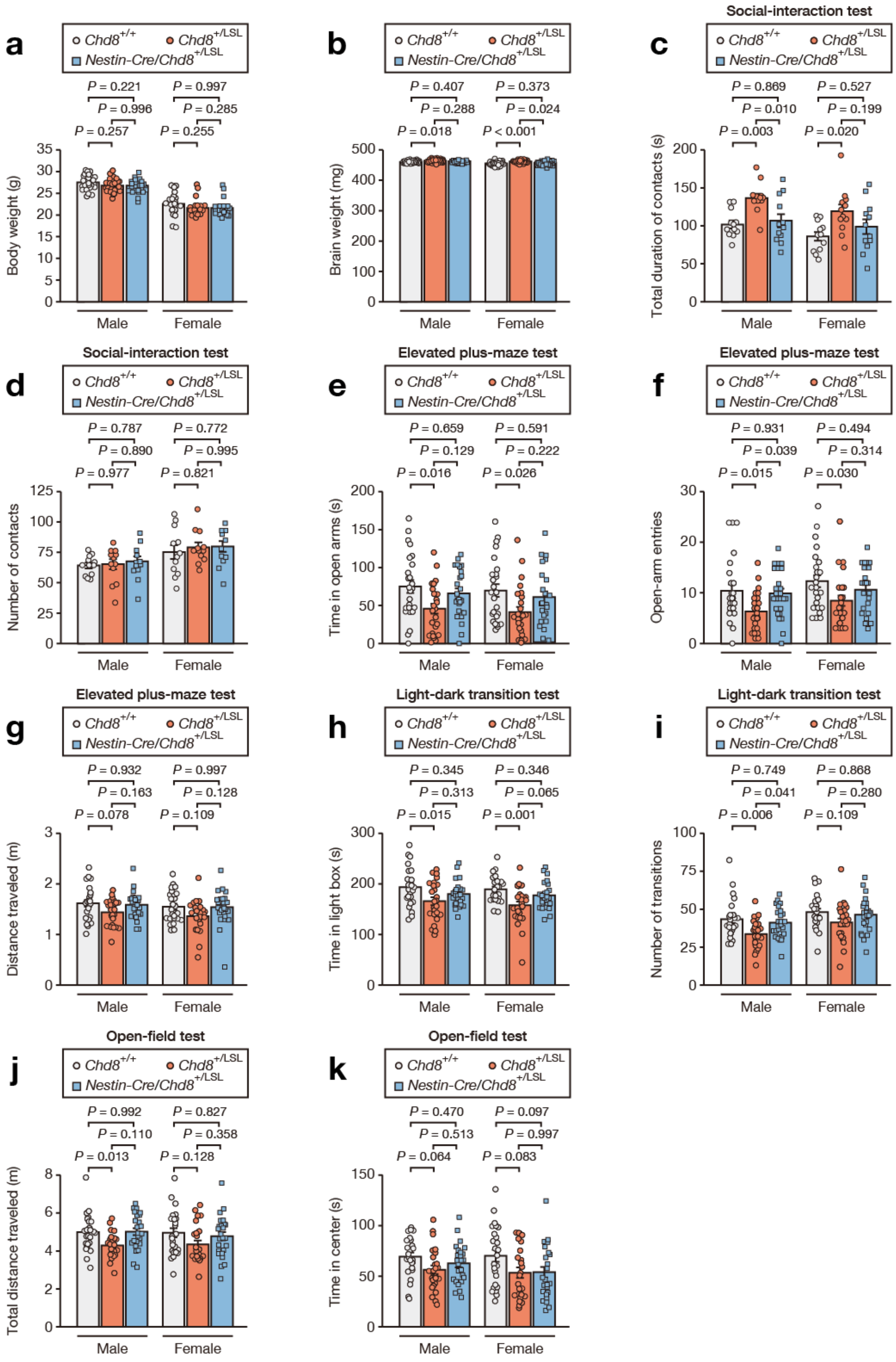

Social-interaction test
Social-interaction test
Elevated plus-maze test
Elevated plus-maze test
Elevated plus-maze test
Light-dark transition test
Light-dark transition test
Open-field test
Open-field test
Chd8+/+
Chd8+/LSL
Nestin-Cre/Chd8+/LSL
Body weight (g)
Brain weight (mg)
Total duration of contacts (s)
Number of contacts
Time in open arms (s)
Open-arm entries
Distance traveled (m)
Time in light box (s)
Number of transitions
Total distance traveled (m)
Time in center (s)
Male
Female

Supplementary Fig. 12 Genetic rescue of *Chd8* expression in neural stem cells ameliorates behavioral abnormalities. **a**,**b**, Body weight (**a**) and brain weight (**b**) for mice subjected to behavioral tests. **c**,**d**, Total duration of contacts (**c**) and total number of contacts (**d**) for the social-interaction test. **e**–**g**, Time spent in the open arms (**e**), number of entries into the open arms (**f**), and total distance traveled (**g**) for the elevated plus-maze test. **h**,**i**, Time spent in the light chamber (**h**) and number of transitions between the light and dark chambers (**i**) for the light-dark transition test. **j**,**k**, Total distance traveled (**j**) and time spent in the central area (**k**) for the open-field test. All data are means ± s.e.m. and were obtained with $Chd8^{+/+}$, $Chd8^{+/LSL}$, and $Nestin\text{-}Cre/Chd8^{+/LSL}$ males (n = 25 mice per genotype, with the exception that n = 12 pairs of mice for the social-interaction test) and females (n = 25 mice per genotype, with the exception that n = 12 pairs of mice for the social-interaction test). All behavioral tests were conducted with mice at 9 to 13 weeks of age. P values were calculated by one-way ANOVA with Tukey's post hoc test.

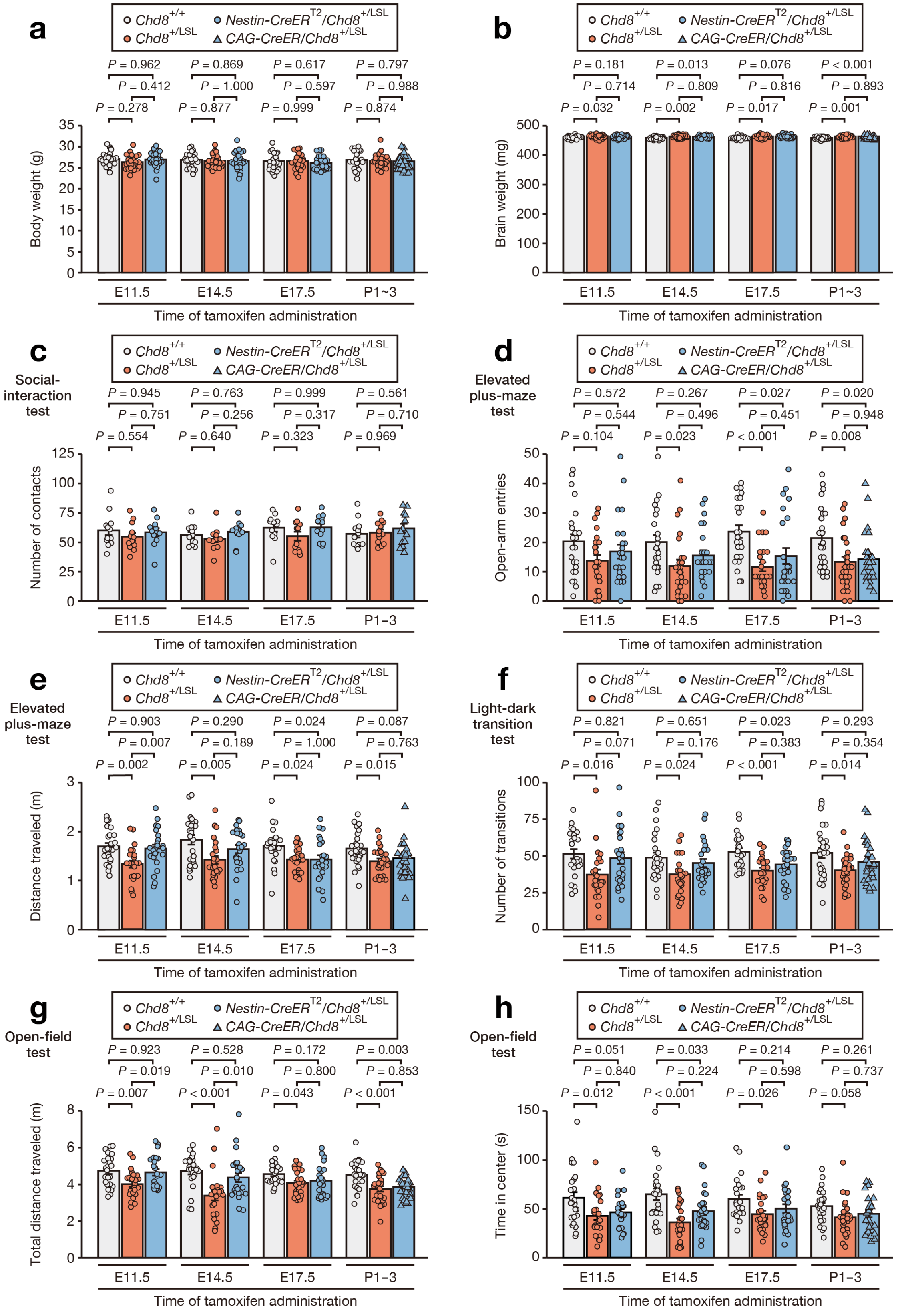
a
Chd8+/+ Chd8+/LSL Nestin-CreERT2/Chd8+/LSL CAG-CreER/Chd8+/LSL
P = 0.962 P = 0.869 P = 0.617 P = 0.797
P = 0.412 P = 1.000 P = 0.597 P = 0.988
P = 0.278 P = 0.877 P = 0.999 P = 0.874
Body weight (g)
E11.5 E14.5 E17.5 P1~3
Time of tamoxifen administration
b
Chd8+/+ Chd8+/LSL Nestin-CreERT2/Chd8+/LSL CAG-CreER/Chd8+/LSL
P = 0.181 P = 0.013 P = 0.076 P < 0.001
P = 0.714 P = 0.809 P = 0.816 P = 0.893
P = 0.032 P = 0.002 P = 0.017 P = 0.001
Brain weight (mg)
E11.5 E14.5 E17.5 P1~3
Time of tamoxifen administration
c
Social-interaction test
Chd8+/+ Chd8+/LSL Nestin-CreERT2/Chd8+/LSL CAG-CreER/Chd8+/LSL
P = 0.945 P = 0.763 P = 0.999 P = 0.561
P = 0.751 P = 0.256 P = 0.317 P = 0.710
P = 0.554 P = 0.640 P = 0.323 P = 0.969
Number of contacts
E11.5 E14.5 E17.5 P1–3
Time of tamoxifen administration
d
Elevated plus-maze test
Chd8+/+ Chd8+/LSL Nestin-CreERT2/Chd8+/LSL CAG-CreER/Chd8+/LSL
P = 0.572 P = 0.267 P = 0.027 P = 0.020
P = 0.544 P = 0.496 P = 0.451 P = 0.948
P = 0.104 P = 0.023 P < 0.001 P = 0.008
Open-arm entries
E11.5 E14.5 E17.5 P1–3
Time of tamoxifen administration
e
Elevated plus-maze test
Chd8+/+ Chd8+/LSL Nestin-CreERT2/Chd8+/LSL CAG-CreER/Chd8+/LSL
P = 0.903 P = 0.290 P = 0.024 P = 0.087
P = 0.007 P = 0.189 P = 1.000 P = 0.763
P = 0.002 P = 0.005 P = 0.024 P = 0.015
Distance traveled (m)
E11.5 E14.5 E17.5 P1–3
Time of tamoxifen administration
f
Light-dark transition test
Chd8+/+ Chd8+/LSL Nestin-CreERT2/Chd8+/LSL CAG-CreER/Chd8+/LSL
P = 0.821 P = 0.651 P = 0.023 P = 0.293
P = 0.071 P = 0.176 P = 0.383 P = 0.354
P = 0.016 P = 0.024 P < 0.001 P = 0.014
Number of transitions
E11.5 E14.5 E17.5 P1–3
Time of tamoxifen administration
g
Open-field test
Chd8+/+ Chd8+/LSL Nestin-CreERT2/Chd8+/LSL CAG-CreER/Chd8+/LSL
P = 0.923 P = 0.528 P = 0.172 P = 0.003
P = 0.019 P = 0.010 P = 0.800 P = 0.853
P = 0.007 P < 0.001 P = 0.043 P < 0.001
Total distance traveled (m)
E11.5 E14.5 E17.5 P1–3
Time of tamoxifen administration
h
Open-field test
Chd8+/+ Chd8+/LSL Nestin-CreERT2/Chd8+/LSL CAG-CreER/Chd8+/LSL
P = 0.051 P = 0.033 P = 0.214 P = 0.261
P = 0.840 P = 0.224 P = 0.598 P = 0.737
P = 0.012 P < 0.001 P = 0.026 P = 0.058
Time in center (s)
E11.5 E14.5 E17.5 P1–3
Time of tamoxifen administration

Supplementary Fig. 13 Genetic rescue of *Chd8* expression in neural stem cells at E11.5 or E14.5 ameliorates behavioral abnormalities in male mice. a,**b**, Body weight (**a**) and brain weight (**b**) for mice subjected to behavioral tests. **c**, Total number of contacts for the social-interaction test. **d**,**e**, Number of entries into the open arms (**d**) and total distance traveled (**e**) for the elevated plus-maze test. **f**, Number of transitions between the light and dark chambers for the light-dark transition test. **g**,**h**, Total distance traveled (**g**) and time spent in the central area (**h**) for the open-field test. All data are means ± s.e.m. and were obtained with *Chd8*$^{+/+}$, *Chd8*$^{+/\mathrm{LSL}}$, and *Nestin-CreER*$^{\mathrm{T2}}$/*Chd8*$^{+/\mathrm{LSL}}$ adult male mice treated with tamoxifen at E11.5 (n = 24 mice per genotype), E14.5 (n = 25 mice per genotype), or E17.5 (n = 24 mice per genotype), and with *Chd8*$^{+/+}$, *Chd8*$^{+/\mathrm{LSL}}$, and *CAG-CreER*/*Chd8*$^{+/\mathrm{LSL}}$ adult male mice treated with tamoxifen at P1–3 (n = 26 mice per genotype). All behavioral tests were conducted with male mice at 9 to 13 weeks of age. For the social-interaction test only, two unfamiliar mice constituted one experimental unit (one pair); thus, n values are indicated as numbers of mice, statistical analyses were performed based on the number of pairs. P values were calculated by one-way ANOVA with Tukey's post hoc test.

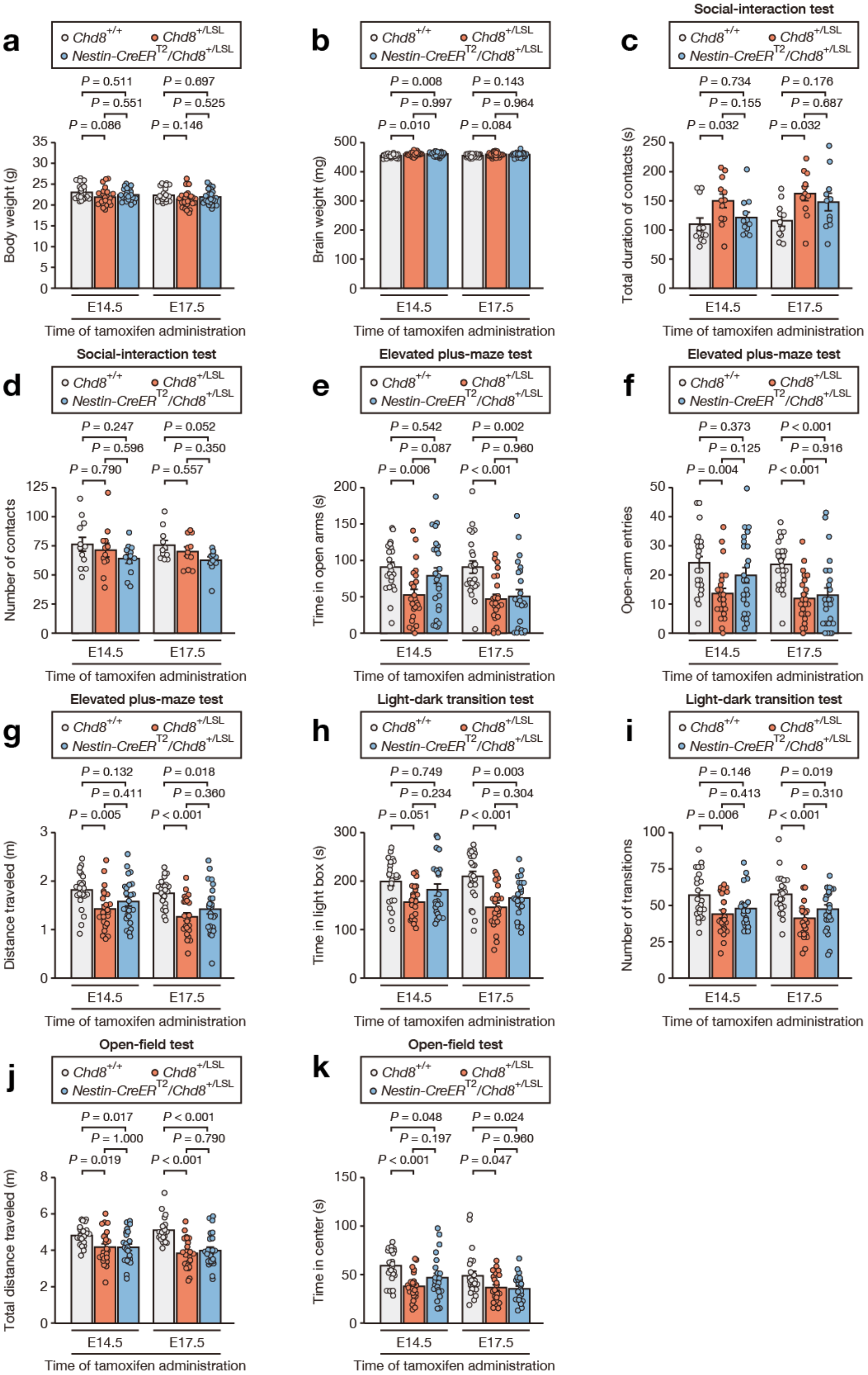
a
Chd8+/+ Chd8+/LSL
Nestin-CreERT2/Chd8+/LSL
P = 0.511 P = 0.697
P = 0.551 P = 0.525
P = 0.086 P = 0.146
Body weight (g)
E14.5 E17.5
Time of tamoxifen administration
b
P = 0.008 P = 0.143
P = 0.997 P = 0.964
P = 0.010 P = 0.084
Brain weight (mg)
c
Social-interaction test
P = 0.734 P = 0.176
P = 0.155 P = 0.687
P = 0.032 P = 0.032
Total duration of contacts (s)
d
Social-interaction test
P = 0.247 P = 0.052
P = 0.596 P = 0.350
P = 0.790 P = 0.557
Number of contacts
e
Elevated plus-maze test
P = 0.542 P = 0.002
P = 0.087 P = 0.960
P = 0.006 P < 0.001
Time in open arms (s)
f
Elevated plus-maze test
P = 0.373 P < 0.001
P = 0.125 P = 0.916
P = 0.004 P < 0.001
Open-arm entries
g
Elevated plus-maze test
P = 0.132 P = 0.018
P = 0.411 P = 0.360
P = 0.005 P < 0.001
Distance traveled (m)
h
Light-dark transition test
P = 0.749 P = 0.003
P = 0.234 P = 0.304
P = 0.051 P < 0.001
Time in light box (s)
i
Light-dark transition test
P = 0.146 P = 0.019
P = 0.413 P = 0.310
P = 0.006 P < 0.001
Number of transitions
j
Open-field test
P = 0.017 P < 0.001
P = 1.000 P = 0.790
P = 0.019 P < 0.001
Total distance traveled (m)
k
Open-field test
P = 0.048 P = 0.024
P = 0.197 P = 0.960
P < 0.001 P = 0.047
Time in center (s)

Supplementary Fig. 14 Genetic rescue of *Chd8* expression in neural stem cells at E14.5 ameliorates behavioral abnormalities in female mice. **a**,**b**, Body weight (**a**) and brain weight (**b**) for mice subjected to behavioral tests. **c**,**d**, Total duration of contacts (**c**) and total number of contacts (**d**) for the social-interaction test. **e–g**, Time spent in the open arms (**e**), number of entries into the open arms (**f**), and total distance traveled (**g**) for the elevated plus-maze test. **h**,**i**, Time spent in the light chamber (**h**) and number of transitions between the light and dark chambers (**i**) for the light-dark transition test. **j**,**k**, Total distance traveled (**j**) and time spent in the central area (**k**) for the open-field test. All data are means ± s.e.m. and were obtained with *Chd8*$^{+/+}$, *Chd8*$^{+/LSL}$, and *Nestin-CreER*^{T2}/*Chd8*$^{+/LSL}$ adult female mice treated with tamoxifen at E14.5 (n = 24 mice per genotype, with the exception that n = 12 pairs of mice for the social-interaction test) or E17.5 (n = 24 mice per genotype, with the exception that n = 12 pairs of mice for the social-interaction test). All behavioral tests were conducted with female mice at 9 to 13 weeks of age. *P* values were calculated by one-way ANOVA with Tukey's post hoc test.

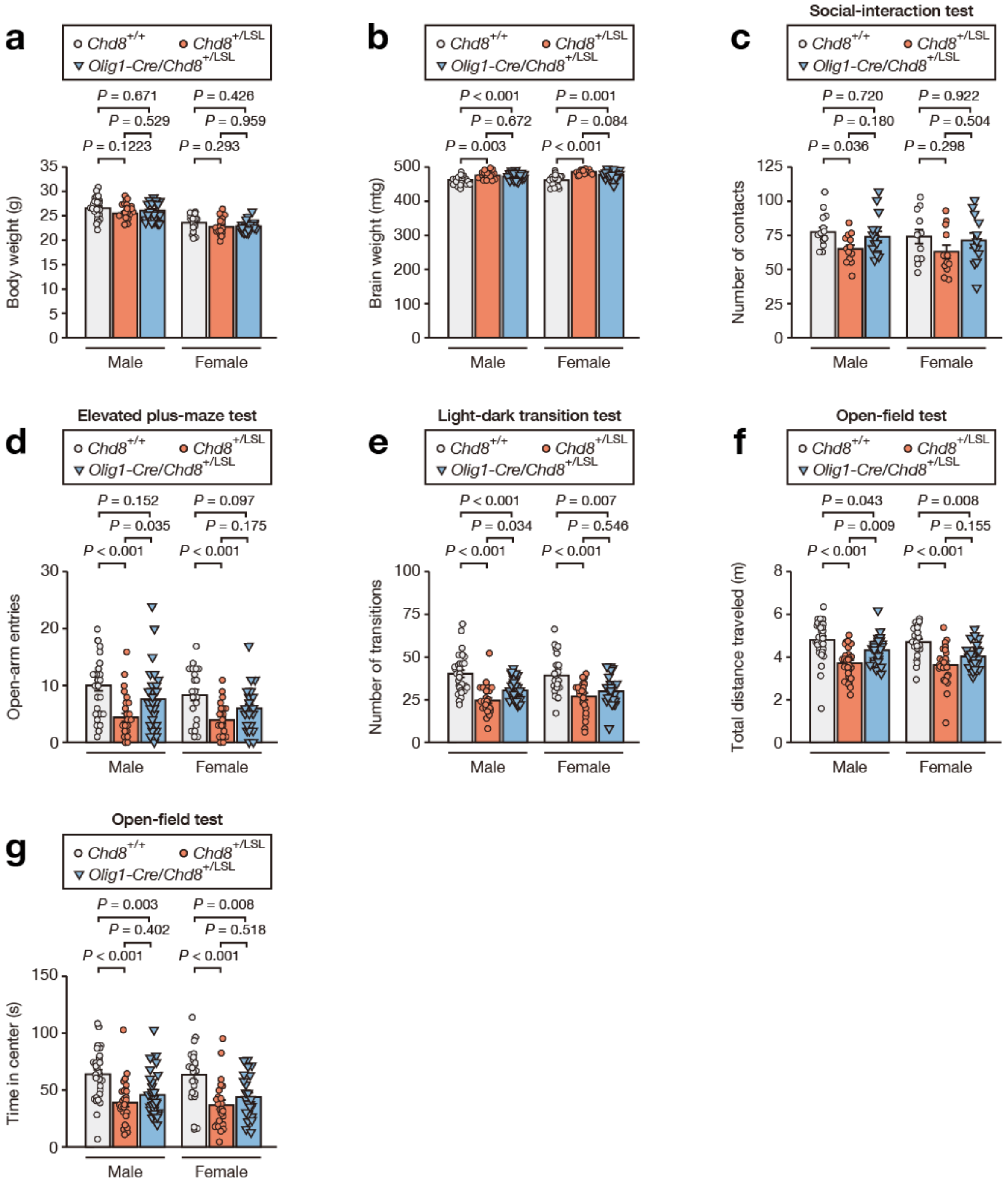

Supplementary Fig. 15 Genetic rescue of *Chd8* expression in ventral progenitor cells ameliorates behavioral abnormalities in male and female mice. a,**b**, Body weight (**a**) and brain weight (**b**) for mice subjected to behavioral tests. **c**, Total number of contacts for the social-

interaction test. **d**, Number of entries into the open arms for the elevated plus-maze test. **e**, Number of transitions between the light and dark chambers for the light-dark transition test. **f**,**g**, Total distance traveled (**f**) and time spent in the central area (**g**) for the open-field test. All data are means ± s.e.m. and were obtained with *Chd8*$^{+/+}$, *Chd8*$^{+/LSL}$, and *Olig1-Cre*/*Chd8*$^{+/LSL}$ adult male mice (n = 30, 29, and 30 mice, respectively) and female mice (n = 24 mice per genotype). For the social-interaction test only, two unfamiliar mice constituted one experimental unit (one pair); thus, n values are indicated as numbers of mice, statistical analyses were performed based on the number of pairs. All behavioral tests were conducted with mice at 9 to 13 weeks of age. P values were calculated by one-way ANOVA with Tukey's post hoc test.
