# Supplementary material for: Defective ventral neurogenesis due to midfetal Chd8 mutation drives autistic-like behavior in mice
Source: Nat Commun. 2026 May 27;17:4457. doi: 10.1038/s41467-026-73416-2 (PMC13216556; doi:10.1038/s41467-026-73416-2)
Supplement: Supplementary file 2 — Description of Additional Supplementary Files [file 41467_2026_73416_MOESM2_ESM.pdf]

Description of Additional Supplementary Files

File name: Supplementary Data 1

Description: Results (fold change and *P* value) for total genes in the scRNA-seq analysis performed in this study.

File name: Supplementary Data 2

Description: List of genes used in the GSEA for the scRNA-seq data in this study.

File name: Supplementary Data 3

Description: Results (fold change and *P* value) for total genes in the spatial transcriptome analysis performed in this study.
