# Supplementary material for: Defective ventral neurogenesis due to midfetal Chd8 mutation drives autistic-like behavior in mice
Source: Nat Commun. 2026 May 27;17:4457. doi: 10.1038/s41467-026-73416-2 (PMC13216556; doi:10.1038/s41467-026-73416-2)
Supplement: Supplementary file 6 — Reporting Summary [file 41467_2026_73416_MOESM6_ESM.pdf]

nature portfolio

Corresponding author(s): Masaaki Nishiyama

Last updated by author(s): Mar 26, 2026

Reporting Summary

Nature Portfolio wishes to improve the reproducibility of the work that we publish. This form provides structure for consistency and transparency in reporting. For further information on Nature Portfolio policies, see our Editorial Policies and the Editorial Policy Checklist.

Statistics

For all statistical analyses, confirm that the following items are present in the figure legend, table legend, main text, or Methods section.

n/a	Confirmed	
☐	☒	The exact sample size (*n*) for each experimental group/condition, given as a discrete number and unit of measurement
☐	☒	A statement on whether measurements were taken from distinct samples or whether the same sample was measured repeatedly
☐	☒	The statistical test(s) used AND whether they are one- or two-sided<br>*Only common tests should be described solely by name; describe more complex techniques in the Methods section.*
☐	☒	A description of all covariates tested
☐	☒	A description of any assumptions or corrections, such as tests of normality and adjustment for multiple comparisons
☐	☒	A full description of the statistical parameters including central tendency (e.g. means) or other basic estimates (e.g. regression coefficient) AND variation (e.g. standard deviation) or associated estimates of uncertainty (e.g. confidence intervals)
☐	☒	For null hypothesis testing, the test statistic (e.g. *F*, *t*, *r*) with confidence intervals, effect sizes, degrees of freedom and *P* value noted<br>*Give P values as exact values whenever suitable.*
☒	☐	For Bayesian analysis, information on the choice of priors and Markov chain Monte Carlo settings
☒	☐	For hierarchical and complex designs, identification of the appropriate level for tests and full reporting of outcomes
☐	☒	Estimates of effect sizes (e.g. Cohen's *d*, Pearson's *r*), indicating how they were calculated

Our web collection on statistics for biologists contains articles on many of the points above.

Software and code

Policy information about availability of computer code

Data collection	Library was prepared using Chromium Next GEM Single Cell 3' Kit v3.1 (10x Genomics). scRNA-seq data were generated with the 10x Genomics Chromium platform. Reads were mapped to the mouse (mm10) genome with the use of Cell Ranger (v7.0.0).<br>For spatial transcriptome analysis, each section was analyzed with the CosMx Spatial Molecular Imager (Nanostring) and a 1000-Plex RNA Mouse Neuroscience Panel by Visualix (Kobe, Japan). Fields of view (FOVs) measuring 0.51 by 0.51 mm were set to include both the cortex and striatum.
Data analysis	Data processing and visualization of scRNA-seq data were performed with Scanpy (v1.9.3). Batch effects were corrected for by application of the single-cell Variational Inference (scVI) model. Gene Ontology analysis of DEGs and GSEA were performed with the use of DAVID and GSEA software (v4.3.2), respectively. scVelo (v0.2.3) was applied to estimate RNA velocity, and cell fate probabilities were estimated with CellRank (v2.0.4).<br>Spatial transcriptomic data were processed using the Scanpy (v1.9.3) and Squidpy (v1.2.4) Python packages. Cell-cell communication analysis was conducted with the NeuronChat R package. The codes for the scRNA-seq and spatial transcriptome analyses used in this study can be found on GitHub: https://github.com/akwamura/Chd8_scRNA-seq, and archived on Zenodo as Kawamura A (2024) Chd8_scRNA-seq: https://doi.org/10.5281/zenodo.19105439.

For manuscripts utilizing custom algorithms or software that are central to the research but not yet described in published literature, software must be made available to editors and reviewers. We strongly encourage code deposition in a community repository (e.g. GitHub). See the Nature Portfolio guidelines for submitting code & software for further information.

Data

Policy information about availability of data

All manuscripts must include a data availability statement. This statement should provide the following information, where applicable:

- Accession codes, unique identifiers, or web links for publicly available datasets
- A description of any restrictions on data availability
- For clinical datasets or third party data, please ensure that the statement adheres to our policy

scRNA-seq data have been deposited in GEO under the accession number GSE278323 and in DDBJ Sequence Read Archive under the accession number DRA016611. Spatial transcriptome analysis data have been deposited in GEO under the accession number GSE278133.

Research involving human participants, their data, or biological material

Policy information about studies with human participants or human data. See also policy information about sex, gender (identity/presentation), and sexual orientation and race, ethnicity and racism.

Reporting on sex and gender	NA
Reporting on race, ethnicity, or other socially relevant groupings	NA
Population characteristics	NA
Recruitment	NA
Ethics oversight	NA

Note that full information on the approval of the study protocol must also be provided in the manuscript.

Field-specific reporting

Please select the one below that is the best fit for your research. If you are not sure, read the appropriate sections before making your selection.

[x] Life sciences [ ] Behavioural & social sciences [ ] Ecological, evolutionary & environmental sciences

For a reference copy of the document with all sections, see nature.com/documents/nr-reporting-summary-flat.pdf

Life sciences study design

All studies must disclose on these points even when the disclosure is negative.

Sample size	Although no statistical power analysis was performed beforehand, our sample sizes were in accordance with those generally adopted in the field.
Data exclusions	No data were excluded from the analyses.
Replication	The experimental findings were reliably reproduced through repeated experiments.
Randomization	All individual mice or samples were randomly allocated to the experimental groups.
Blinding	The experimenters were blinded to mouse genotype during data collection.

Reporting for specific materials, systems and methods

We require information from authors about some types of materials, experimental systems and methods used in many studies. Here, indicate whether each material, system or method listed is relevant to your study. If you are not sure if a list item applies to your research, read the appropriate section before selecting a response.

Materials & experimental systems

n/a	Involved in the study	
	x	Antibodies
x		Eukaryotic cell lines
x		Palaeontology and archaeology
	x	Animals and other organisms
x		Clinical data
x		Dual use research of concern
x		Plants

Methods

n/a	Involved in the study	
x		ChIP-seq
	x	Flow cytometry
x		MRI-based neuroimaging

Antibodies

Antibodies used	Antibodies included those to SOX2 (ab97959, abcam; 1:1000 dilution), Ki67 (550609, BD Biosciences; 1:500), DCX (ab18723, abcam; 1:1000), OLIG2 (AB9610, Millipore; 1:500), DLX1 (Af460, Frontier Institute; 1:500), PDGFRa (558774, BD Pharmingen; 1:500), GABA (A2052, Sigma-Aldrich; 1:500), vGAT (14471-1-AP, proteintech; 1:500), and NeuN (MAB377, Millipore; 1:1000) for immunofluorescence staining, and those to CHD8 (77694, Cell Signaling Technology; 1:1000) and HSP90 (610419, BD Biosciences; 1:1000) for immunoblot analysis. Antibodies to RFP (5f8, ChromoTek; or MA5-15257, Thermo Fisher Scientific, both at 1:1000) were used to enhance the tdTomato signal in immunofluorescence analysis.
Validation	Antibody validation was reported from the companies that provide the antibodies or from cited literature.

Animals and other research organisms

Policy information about studies involving animals; ARRIVE guidelines recommended for reporting animal research, and Sex and Gender in Research

Laboratory animals	The generation of Chd8+/F mice was described previously (Katayama, et al., Nature. 2016). Offspring were backcrossed onto the C57BL/6J line for at least nine generations. Chd8+/F mice were crossed with Nestin-Cre, Nestin-CreERT2, or CAG-CreER heterozygous mice to produce Nestin-Cre/Chd8+/F, Nestin-CreERT2/Chd8+/F, or CAG-CreER/Chd8+/F mice, respectively.<br>Chd8 conditional knock-in floxed (Chd8+/LSL) mice were generated by CRISPR/Cas9-based genome editing in the C57BL/6J strain. The donor plasmid contained an adenoviral splice acceptor site and a stop cassette flanked by loxP sequences.<br>Vgat-ires-Cre mice were generated by in vitro fertilization using cryopreserved sperm provided by Dr. K. Kaneda at Kanazawa University. The original mice were purchased from The Jackson Laboratory (STOCK Slc32a1tm2(cre)Lowl/J, Strain #:016962).<br>Mice were housed under a 12-h light/dark cycle at 23±3°C and 55±10% humidity, with ad libitum access to food and water.
Wild animals	NA
Reporting on sex	Both sexes were used in this study. For postnatal experiments, the sex of mice was identified and specified in the figure legends where applicable. For fetal experiments, sex was not determined due to technical limitations at the embryonic stage. For scRNA-seq and transcriptome analysis, we used samples from male mice, as previous studies primarily employed male mice, and we aimed to compare our results with those studies.
Field-collected samples	NA
Ethics oversight	All experiments were performed with the approval of the Animal Care Committee of Kanazawa University (protocol no. KINDAI 6-2135).

Note that full information on the approval of the study protocol must also be provided in the manuscript.

Plants

Seed stocks	NA
Novel plant genotypes	NA
Authentication	NA

Flow Cytometry

Plots

Confirm that:

- [x] The axis labels state the marker and fluorochrome used (e.g. CD4-FITC).
- [x] The axis scales are clearly visible. Include numbers along axes only for bottom left plot of group (a 'group' is an analysis of identical markers).
- [x] All plots are contour plots with outliers or pseudocolor plots.
- [x] A numerical value for number of cells or percentage (with statistics) is provided.

Methodology

Sample preparation	The telencephalon including the cortex and striatum (without the olfactory bulb and thalamus) was collected from P5 or embryonic mice harboring Nestin-CreERT2/Rosa26-tdTomato after tamoxifen treatment. The tissue was dissociated with a papain dissociation system (LK003150, Worthington Biochemical).
Instrument	FACS was performed with a FACSAria Fusion Cell Sorter (BD Biosciences) fitted with a 100-µm nozzle.
Software	The data generated during cell sorting were analyzed with FlowJo_V10 software.
Cell population abundance	Approximately 25% of input cells were sorted as tdTomato positive after gating for forward/side scatter, LIVE/DEAD dye (Pacific Blue), and tdTomato (phycoerythrin) fluorescence.
Gating strategy	After gating for forward and side scatter, approximately 85% of cells were sorted. Then approximately 15% of cells labeled with the LIVE/DEAD dye were removed by gating for Pacific Blue fluorescence. Finally about one-third of cells were sorted as tdTomato-positive by gating for phycoerythrin fluorescence.

- [x] Tick this box to confirm that a figure exemplifying the gating strategy is provided in the Supplementary Information.
